# Quality of life study of patients with unresectable locally advanced or metastatic pancreatic adenocarcinoma treated with gemcitabine+nab-paclitaxel versus gemcitabine alone: AX-PANC-SY001, a randomized phase-2 study

**DOI:** 10.1186/s12885-020-06758-9

**Published:** 2020-03-30

**Authors:** Suayib Yalcin, Faysal Dane, Berna Oksuzoglu, Nuriye Yildirim Ozdemir, Abdurrahman Isikdogan, Metin Ozkan, Guzin Gonullu Demirag, Hasan Senol Coskun, Bulent Karabulut, Turkkan Evrensel, Mehmet Ali Ustaoglu, Feyyaz Ozdemir, Hande Turna, Tugba Yavuzsen, Faruk Aykan, Alper Sevinc, Hakan Akbulut, Deniz Yuce, Mutlu Hayran, Saadettin Kilickap

**Affiliations:** 1 Hacettepe University Faculty of Medicine, Ankara, Turkey; 2 Hacettepe University Cancer Institute, Ankara, Turkey; 3 Marmara University Faculty of Medicine, İstanbul, Turkey; 4 Dr. Abdurrahman Yurtaslan Ankara Oncology Training and Research Hospital, Ankara, Turkey; 5 Ankara Numune Training and Research Hospital, Ankara, Turkey; 6 Dicle University Faculty of Medicine, Dyarbakir, Turkey; 7 Erciyes University Faculty of Medicine, Kayseri, Turkey; 8 Ondokuz Mayis University Faculty of Medicine, Samsun, Turkey; 9 Akdeniz University Faculty of Medicine, Antalya, Turkey; 10 Ege University Faculty of Medicine, İzmir, Turkey; 11 Uludag University Faculty of Medicine, Bursa, Turkey; 12 Lütfi Kirdar Kartal Training and Research Hospital, İstanbul, Turkey; 13 Karadeniz Teknik University Faculty of Medicine, Trabzon, Turkey; 14 İstanbul University Cerrahpasa Faculty of Medicine, Bursa, Turkey; 15 Dokuz Eylül University Faculty of Medicine, İzmir, Turkey; 16 İstanbul University Cancer Institute, İstanbul, Turkey; 17 Gaziantep University Faculty of Medicine, Gaziantep, Turkey; 18 Ankara University Faculty of Medicine, Ankara, Turkey

**Keywords:** Nab-paclitaxel, Gemcitabine, Pancreatic cancer, Metastatic, Quality of life

## Abstract

**Background:**

Combination of gemcitabine and nab-paclitaxel has superior clinical efficacy than gemcitabine alone. Nevertheless, health-related quality of life. (QoL) associated with this combination therapy when administered at first-line in advanced pancreatic adenocarcinoma is unknown.

**Methods:**

A total of 125 patients were randomized to combination therapy (1000 mg/m2 gemcitabine + 125 mg/m2 nab-paclitaxel) and single-agent gemcitabine (1000 mg/m2) arms to take treatment weekly for 7 of 8 weeks, and following 3 of 4 weeks, until progression or severe toxicity. Primary endpoints were three-months of definitive deterioration free percent of patients, and QoL.

**Results:**

Overall QoL analyses showed that 34 and 58.3% of cases in gemcitabine and gemcitabine+nab-P arms had no deterioration in 3rd month QoL scores (*p* = 0.018). These proportions were 27.3 and 36.6% in 6^th^ month assessments, respectively (*p* = 0.357). Median overall survivals in combination and single-agent arms were 9.92 months and 5.95 months, respectively (HR: 0.64, 95% CI: 0.42–0.86, *p* = 0.038). Median progression free survivals in these treatment arms were 6.28 and 3.22 months, respectively (HR: 0.58, 95% CI: 0.39–0.87, *p* = 0.008). Median time-to-deterioration were 5.36 vs 3.68 months, and objective response rates were 37.1% vs 23.7% (*p* = 0.009), respectively in combination and single-agent arms.

**Conclusions:**

Combination therapy with gemcitabine + nab-paclitaxel had better overall and progression-free survival than gemcitabine alone. Also, combination therapy showed increased response rate without toxicity or deteriorated QoL. Combination treatment with gemcitabine and nab-paclitaxel may provide significant benefit for advanced pancreatic cancer.

**Trial registration:**

This study has been registered in ClinicalTrials.gov as NCT03807999 on January 8, 2019 (retrospectively registered).

## Background

Approximately forty-thousand patients with pancreatic cancer die annually, which corresponds to 4^th^ most common cancer-caused deaths when both sexes combined. The incidence of pancreatic cancer has tripled since 1950s, nevertheless this sharp increase ranked pancreatic carcinoma only to the 10^th^ most common cancer regarding incidence rates. The significant difference between incidence and mortality rankings is associated with poor disease prognosis (mortality/incidence ratio: 98%) [1]. The 5-year overall survival (OS) rate is approximately 4%. Current evidence suggests that locally advanced or metastatic disease poorly responds to chemotherapy. When compared to fluorouracil, gemcitabine may modestly improve survival, but median OS in advanced cases still below 6 months [2]. Previous randomized phase-III studies showed no significant OS difference between cytotoxic drug combinations and gemcitabine-only regimens.

Randomized phase III PRODIGE trial evaluated FOLFIRINOX regimen in metastatic pancreatic cancer patients [3]. Both median progression-free survival (PFS) (6.4 vs 3.3 months, *p* < 0.001) and median OS (11.1 vs 6.8 months, *p* < 0.001) were dramatically improved. In patients with good performance status FOLFIRINOX remains a viable first-line option. However, toxicity of FOLFIRINOX regimen still remains a concern.

The effect of FOLFIRINOX on quality of life (QoL) in metastatic pancreatic cancer was analyzed from the PRODIGE 4/ACCORD 11 trial [4]. FOLFIRINOX combination was found to significantly reduce QoL impairment compared with single-agent gemcitabine. Moreover, incorporation of baseline QoL scores to clinical and demographic data showed better survival probabilities.

The albumin-bound paclitaxel, namely nab-paclitaxel (nab-P), is a particular nanoparticle form of paclitaxel. The phase-III MPACT trial compared nab-P with gemcitabine in 861 patients with metastatic pancreatic adenocarcinoma. In this study, the nab-P arm received 125 mg/m2 of nab-p then 1000 mg/m2 gemcitabine for 3 weeks followed by a week of rest, and gemcitabine arm received 1000 mg/m2 of gemcitabine monotherapy for 7 weeks followed by a week of rest, and then weekly gemcitabine for 3 weeks plus 1 week of rest [5]. Authors reported that OS was significantly improved in nab-P arm (median 8.5 mo) compared to gemcitabine monotherapy (median 6.7 mo) (HR = 0.72; *p* < 0.001), which suggests a 31% reduction in the risk of progression or death. Twelve-month survival rates were 35% vs 22% in combination and gemcitabine-only regimens, respectively, which suggests a 59% increase in survival (*p* = 0.0002). Moreover, median PFSs were 5.5 vs 3.7 months (HR: 0.69; *p* = 0.000024), and overall response rates were 23% vs 7%, respectively, which all favors combination treatment. The toxicity of the combination was modest and easily manageable. This combination may represent a new standard in the management of these patients.

QoL changes in patients receiving nab-P in combination with gemcitabine for the first-line treatment of metastatic or locally advanced unresectable pancreatic adenocarcinoma have not been explored. This randomized, phase II study analyzes the effect of nab-P plus gemcitabine on QoL of these patients. Efficacy and safety of the combination will also be analyzed. The randomized phase-III MPACT trial showed that gemcitabine + nab-P combination has superior clinical efficacy than gemcitabine-only regimen, but QoL associated with combination regimen at first-line in unresectable locally advanced or metastatic pancreatic ductal adenocarcinoma is still unknown.

## Methods

This study included a total of 125 patients ≥18 years-old and presented with metastatic or unresectable pancreatic adenocarcinoma and without prior chemotherapy. Twenty-three patients (18.4%) had locally advanced disease, and 102 patients (81.6%) had metastatic disease. Other inclusion criteria were having a measurable/evaluable disease by RECIST, ECOG performance status 0 or 1, adequate bone marrow functions (granulocyte count ≥1500/mm^3^, platelet count ≥100,000/mm^3^), and adequate liver functions (Total biliribin < 2 mg/dL, ALP/GGT < 5 x upper normal limit – *UNL*, ALT/AST < 2.5 x UNL). The study protocol was approved by the Malatya Clinical Trials Ethical Committee of the Inonu University on 21 May 2014, and a signed informed consent were obtained from all patients. Exclusion criteria were as follows:

- Patients over 76 years-old, with active infection or chronic diarrhea
- Any prior treatment for metastatic pancreatic cancer. Only exception is systemic adjuvant treatment with/without radiation that completed > 6 months before enrollment
- Being unable to comply to protocol
- Presence of severe cardiac disease including but not limited to congestive heart failure, symptomatic coronary artery disease, uncontrolled cardiac arrhythmias, or myocardial infarction within the last 12 months
- Presence of any other major organ failure, or metastases in central nervous system
- Expert opinion about increased risk of treatment or possibility of getting misleading results that might bias the study
- Very poor life expectancy that < 12 weeks
- Pregnancy (positive pregnancy test) or lactation
- Prior malignancy other than skin cancer (basal cell), in-situ cervical cancer, any well-treated Stage-I/II cancer with complete remission or disease-free status more than 5 years
- Physically disintegrated upper gastro-intestinal tract, or presence of any malabsorption syndrome
- Uncontrolled coagulopathy, or concurrent/pre-existing coumadine use
- Sensory neuropathy > grade 1.
- Major surgery without complete recovery within 4 weeks of the study commencement

Following recruitment, patients were 1:1 randomized to receive gemcitabine+nab-P, or gemcitabine alone. Treatment continued until disease progression or unacceptable toxicity.

Patients were treated on an outpatient basis with nab-P + gemcitabine combination or single-agent gemcitabine. Patients receiving nab-P + gemcitabine received 30–40 min infusion of 125 mg/m^2^ nab-P (max 40 min) followed by 30–40 min infusion of 1000 mg/m^2^ gemcitabine (max 40 min) for 3 weeks, followed by a week of rest.

Patients receiving gemcitabine alone received weekly 30–40 min infusion of 1000 mg/m^2^ gemcitabine (max 40 min) for 7 weeks, followed by a week of rest (8-week cycle; Cycle 1 only), followed by cycles of weekly administration for 3 weeks (on Days 1, 8, and 15) followed by one week of rest (4-week cycle). Available therapies were given for the 2nd line treatment and supportive care on investigator’s discretion.

Primary endpoint was 3-month deterioration free rate (percentage of patients free from definitive deterioration). The time until definitive deterioration (TUDD) was calculated using the time that an at least 10 point of decrease in EORTC QLQ-C30 scores has been observed. The quality of life assessments were also compared between study arms. The QLQ-C30 assessments were done every 4 weeks based on recommendations from EORTC.

Secondary endpoints were overall survival, progression free survival, and response rate.

### EORTC QLQ-C30 questionnaire

The QoL of patients was assessed using the health-related quality of life questionnaire for cancer patients that developed by the European Organization for Research and Treatment (EORTC), the QLQ-C30 questionnaire. This scale included 30 items, which evaluate the symptoms and functions of the patients in 5 function domains as physical, role, social, emotional and cognitive functioning; 9 symptom domains as pain, fatigue, financial impact, appetite loss, nausea/vomiting, diarrhea, constipation, sleep disturbance; and, an overall QoL score that reflects the global health status.

### Statistical analysis

Descriptive statistics were presented with mean or median for numerical variables, and frequency and percent for categorical variables. Regarding comparisons between treatment arms, the numerical data including EORTC-QLQ-C30 scores were compared using Mann-Whitney U test, and the categorical data including proportions of patients with and without deterioration in QoL were compared using Chi-square test. Survival analyses regarding the TUDD were conducted with Kaplan-Meier method, and comparisons of survival curves were analyzed with log-rank test. SPSS 21 (IBM Inc., Armonk, NY, USA) software was used for the statistical analyses of the study.

## Results

A total of 125 patients were included in the study. Median ages of the patients in gemcitabine arm were significantly younger than the patients in gemcitabine+nab-P arm (*p* = 0.031), but sex distribution were similar in both groups (M/F_gemcitabin_: 38/25; M/F_gemcitabine + nab-P_: 38/24; *p* = 0.911). Forty percent of disease localization in gemcitabine arm and 45% in gemcitabine+nab-P arm were at pancreatic head, and distributions of disease localization were similar between study arms (*p* = 0.325). Comparison of treatment response between arms revealed a significant difference (*p* = 0.009), which caused by the progressive disease in gemcitabine arm (42.4%) versus gemcitabine+nab-P arm (19.4%). General characteristics of patients were summarized in Table 1.

**Table 1:** General demographics and clinical characteristics of study arms

	**Gemcitabine**	**Gemcitabine + nab-P**	**p**
	**Median [range]**	**Median [range]**	
**Age (years)**	65.5 [37–78]	62 [26–76]	0. 031
	**n (%)**	**n (%)**	**p**
**Sex**			0. 911
***Male***	38 (60.3)	38 (61.3)	
***Female***	25 (39.7)	24 (38.7)	
**Localization**			0. 325
***Head***	25 (40.3)	27 (45)	
***Head + corpus***	8 (12.9)	4 (6.7)	
***Corpus***	11 (17.7)	11 (18.3)	
***Corpus + tail***	5 (8.1)	7 (11.7)	
***Tail***	7 (11.3)	10 (16.7)	
**Response**			0. 009*
***CR***	2 (3.4)	–	
***PR***	12 (20.3)	23 (37.1)	
***SD***	20 (33.9)	27 (43.5)	
***PD***	25 (42.4)	12 (19.4)	

*Rate of progressive disease is responsible from the difference

Regarding the primary endpoint of the study, overall QoL analyses showed that 34 and 58.3% of cases in gemcitabine and gemcitabine+nab-P arms had no deterioration in 3rd month QoL scores (*p* = 0.018). These proportions were 27.3 and 36.6% in 6th month assessments, respectively (*p* = 0.357).

The QoL assessments are presented in Table 2. Percent changes in gemcitabine and gemcitabine+nab-P arms at 3rd month assessments revealed that functional scales of EORTC-QLQ-C30 scale were significantly improved in gemcitabine+nab-P arm, whereas these were deteriorated in gemcitabine arm. The percent changes in the symptom scales were similar between study arms, but the fatigue score increased more in gemcitabine arm significantly, which is related with increased fatigue in patients receiving gemcitabine. The 6th month QoL assessments revealed that the percent changes in both study arms from the previous assessment were not statistically different. All patients completed the QoL questionnaires during treatment period without attrition, and there was no differential compliance between treatment arms that might affect the results of QoL assessments.

**Table 2:** QoL assessments at 3^rd^ and 6^th^ months

	3^rd^ month QoL change %			6^th^ month QoL change %		
	Gemcitabine	Gemcitabine + nab-Paclitaxel	p	Gemcitabine	Gemcitabine + nab-Paclitaxel	p
	Mean	Mean		Mean	Mean	
**Functional scales**						
***Cognitive functioning***	−8.8	23.8	0.048	21	22.5	0.387
***Emotional functioning***	12.9	50.8	0.048	0.6	37.8	0.851
***Physical functioning***	−11.7	12.8	0.051	−4.9	5.7	0.761
***Role functioning***	2.9	17.4	0.039	42.3	10.9	0.456
***Social functioning***	−1.6	30.4	0.024	7.5	3.9	0.743
**Symptom scales**						
***Appetite loss***	−12.5	−14.4	0.226	−13.3	− 15	0.771
***Constipation***	14.1	25.9	0.706	22.9	8.6	0.888
***Diarrhea***	2.6	17.6	0.558	16.9	−1.4	0.707
***Dyspnea***	−32.6	−7.4	0.172	11	−17.6	0.206
***Fatigue***	60.4	5.9	0.027	−51.4	−30.4	0.924
***Financial difficulties***	−11	12.1	0.077	−13.8	12.8	0.879
***Insomnia***	−19	11	0.141	−9.6	7.7	0.276
***Nausea-vomiting***	−4.1	12	0.559	−0.6	−0.8	0.898
***Pain***	−13.8	12.4	0.142	−11.3	− 16.6	0.514
***Global health status/QoL***	12.7	8	0.163	70.6	−9.2	0.158

**Fig. 1:**
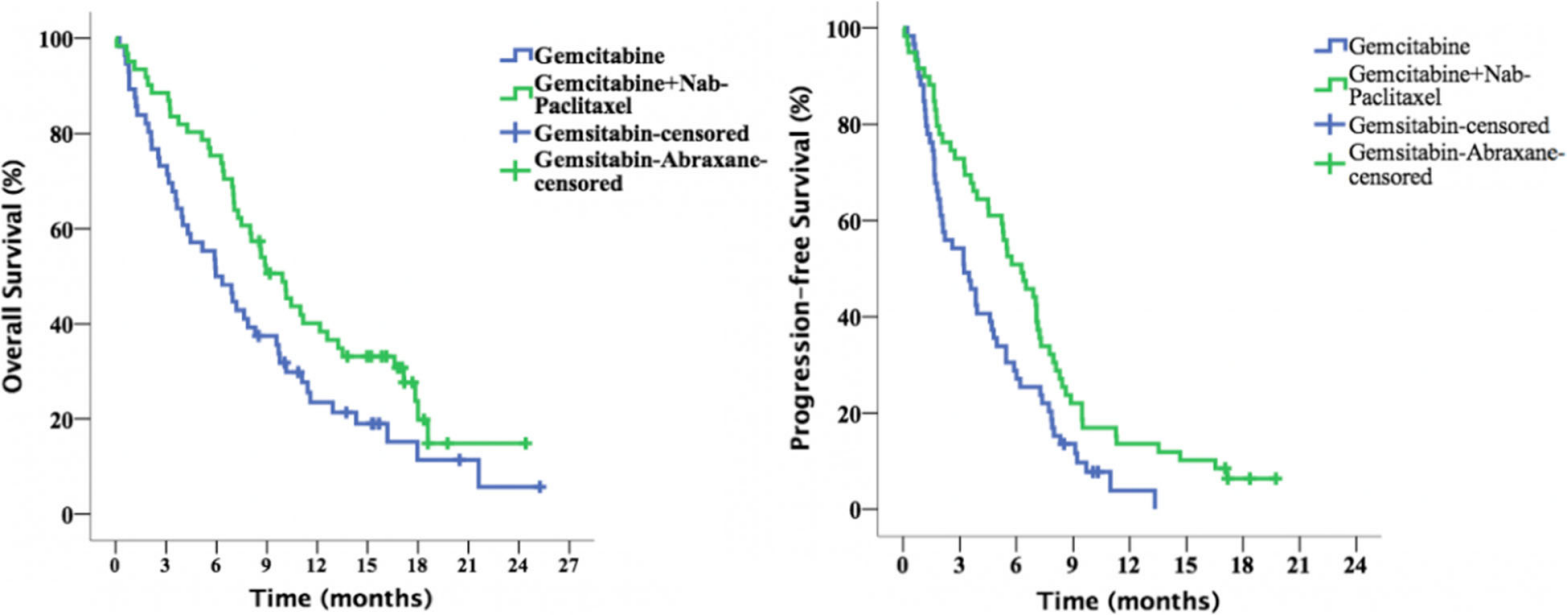
Overall and progression-free survival

The median overall survival was 9.92 and 5.95 months in the combination and single-agent arms, respectively (HR: 0.642, 95% CI: 0.422 to 0.866, *p* = 0.038). Median PFS was 6.28, and 3.22 months in the respective arms (HR: 0.582, 95% CI: 0.391–0.866, *p* = 0.008). The objective response rate was 37.1% vs 23.7%, respectively, and the difference was statistically significant (*p* = 0.009). And, median TUDD was 5.36 vs 3.68 months, respectively. The overall and progression-free survival in both arms are presented in Table 3 and Fig. 1.

**Table 3:** Overall- and progression-free survival in study arms

	Gemcitabine	Gemcitabine+nab-P	Overall	HR*	95% CI	p
**Median Overall Survival (mo)**	5.95	9.92	8.02	0.642	0.422–0.866	0.038
**Median Progression-free Survival (mo)**	3.22	6.28	4.60	0.582	0.391–0.866	0.008

*Age-adjusted HR for Gemcitabine vs. Gemcitabine + Nab-P  arm

## Discussion

In this randomized, phase II study, we have evaluated the effects of nab-P + gemcitabine on QoL, at the first-line treatment of patients with unresectable locally advanced or metastatic pancreatic ductal adenocarcinoma. At the time of the study conducted, the nab-P was not registered and licensed for use in the Turkey, and this study has also provided an opportunity for the patients to reach to this treatment option. As an overall interpretation of our results, when compared with gemcitabine only, gemcitabine+nab-P was associated with an overall and progressive free survival advantage, with increased response rate, without increasing toxicity and deterioration of quality of life. Although these are promising results about treatment associated QoL of patients, one may still have concerns about including both unresectable locally advanced and metastatic patients in the analyses as a common group, which might be seen as a confounding factor to interpret the outcomes. But, since the proportion of unresectable locally advanced patients are less than one fifth of the total population, and since both groups share a common approach regarding treatment in our study, we have not separated the analyses, and think that our results are more generalizable to the treatment of pancreatic cancer patients with advanced disease.

The trajectory of the treatment of advanced pancreatic cancer showed significant survival advantage during its course, but not without sacrificing the QoL of the patients. The primary objective of drug development is to improve patient survival, and this applies to the clinical trials in advanced pancreatic cancer. In line with this aim, first effective treatment for pancreatic cancer has emerged as gemcitabine in 1997 [2]. In 2011, PRODIGE trial revealed that FOLFIRINOX provides significant overall and progression-free survival advantage [3]. And, recently MPACT trial reported that nab-P + gemcitabine combination for the first-line treatment of advanced pancreatic cancer provided promising results as improved overall and progression-free survival in these patients [5]. Currently, among the available therapeutic options for advances pancreatic cancer, data about the QoL of these patients is only available for the FOLFIRINOX regimen, which suggests a deterioration in health-related QoL of these patients [4]. The authors also reported that incorporation of baseline QoL of patients for prediction of survival has prognostic value, which might be used as a stratification factor in further clinical trials. Nevertheless, QoL of patients with advanced pancreatic cancer is not currently being evaluated for treatment decision. Since recent advances in the treatment of these patient group has changed the approach to treatment decision by favoring nab-P + gemcitabine combination, the need for data about the QoL during this treatment has emerged. To the best of our knowledge, this is the first study that evaluated QoL in first-line treatment of advanced pancreatic cancer with Abraxane+Gemcitabine. The rationale behind selecting the EORTC QLQ-C30 scale in this study is about providing a comparable data about the QoL of advanced pancreatic cancer patients with the reference paper, the PRODIGE trial, to infer about the probable QoL benefits of gemcitabine or combination with nab-P.

A recent systematic review has evaluated the effects of chemotherapy on QoL of patients with advanced pancreatic cancer [6]. According to the results of this review, 19 of 23 studies that evaluated the QoL did not report any difference between treatment arms that vary between studies selected. But, 4 studies have reported QoL of the patients with advanced pancreatic cancer showed significant difference between treatment arms. Accordingly, in EORTC QLQ-C30 assessments, global health scores, functional domains including physical, cognitive and role scores, and symptom domains including fatigue scores were found to be better in gemcitabine when compared to BAY12–9566 [7]. In another study that used Functional Assessment of Cancer Therapy-Pancreas (FACT-Pa) QoL questionnaire, gemcitabine and placebo was better than gemcitabine-marimastat combination [8]. Third study revealed that FOLFIRINOX provided favorable outcomes in EORTC QLQ-C30 assessments when compared to gemcitabine, which was reported as definitive degradation of QoL of 31% vs 66%, respectively (HR: 0.47, 95% CI: 0.30 to 0.70, *P* < 0.001) [3]. And the last study revealed that fluorouracil + cisplatin combination provided better QoL outcomes measured by Spitzer’s Quality of Life Index than fluorouracil alone [9]. As can be seen from these studies, one cannot exactly comment on a specific chemotherapeutic agent to provide favorable QoL outcomes than the other agents. Nevertheless, the agents assessed in above studies have some gradual survival advantages to each other, which lacks to be supported by the QoL advantage.

One of the main reasons for heterogeneity in the results of comparison trials about QoL in advanced pancreatic cancer is mainly based on the nature of the disease. Besides the disease itself possess a significant burden of mortality and morbidity on patients, it also causes distressing symptom, which the two most common are pain and cachexia [10, 11]. These two primary factors that tightly associated with pancreatic cancer also has significant contribution to the deterioration of QoL. The variability in the presence of these clinical conditions affect the results of trials that compare the QoL between treatment agents. Since these major symptoms and QoL of the patients are significantly correlated, controlling for the QoL at treatment initiation might be an alternative approach for treatment decision. Moreover, close follow-up of patients for QoL during treatment and appropriate interventions to improve QoL might provide additional advantage for the patient outcomes in advanced pancreatic cancer treatment.

Secondary outcomes in this study were the overall and progression-free survival. Our results revealed that nab-P + gemcitabine arm had significant overall (9.92 vs. 5.95 mo.) and progression-free (6.28 vs. 3.22 mo.) survival advantage when compared to gemcitabine alone. In previous randomized studies that compared nab-P + gemcitabine vs. gemcitabine alone in advanced pancreatic cancer, OS advantage was 2.1 months (median 8.7 vs. 6.6 months) in MPACT trial and 4.8 months (median 11.9 vs. 7.1 months) in the Canadian subgroup analysis of MPACT trial; and PFS advantages were 1.8 months (median 5.5 vs. 3.7 months) and 2 months (median 5.5 vs. 3.7 months) in corresponding studies, respectively. The 4 months of OS advantage and 3 months of PFS advantage in our study are comparable with these previous reports, which suggest similar efficiency of nab-P + gemcitabine in patients with advanced pancreatic cancer. The contribution of QoL advantage in this treatment option also contributes much to the data about the outcomes of this treatment regimen.

## Conclusions

As an overall conclusion of our study, gemcitabine and nab-P combination regimen is a preferable option for the treatment of patients with advanced pancreatic cancer when compared to gemcitabine alone, by means of both survival advantage and also deterioration-free QoL.

## Data Availability

The datasets used and/or analyzed during the current study are available from the corresponding author on reasonable request.

## Abbreviations

ALP: Alkaline phosphatase
ALT: Amino alanine transferase
AST: Aspartate amino transferase
CI: Confidence interval
ECOG: Eastern Cooperative Oncology Group
EORTC: European Organisation for Research and Treatment of Cancer
FACT-Pa: Functional Assessment of Cancer Therapy-Pancreas
GGT: Gamma-glutamyl transpeptidase
nab-P: Nab-paclitaxel
M/F: Male/Female
M/I ratio: Mortality/Incidence ratio
MPACT: Metastatic Pancreatic Adenocarcinoma Clinical Trial
OS: Overall survival
PFS: Progression-free survival
QoL: Quality of life
RECIST: Response Evaluation Criteria In Solid Tumors
TUDD: Time Until Definitive Deterioration
UNL: Upper normal limit
